# Genetic defects in dolichol metabolism

**DOI:** 10.1007/s10545-014-9760-1

**Published:** 2014-10-01

**Authors:** Anna Buczkowska, Ewa Swiezewska, Dirk J. Lefeber

**Affiliations:** 1 Institute of Biochemistry and Biophysics, Polish Academy of Sciences, Pawinskiego 5a, 02-106 Warsaw, Poland; 2 Department of Neurology, Laboratory of Genetic, Endocrine and Metabolic Diseases, Radboud University Medical Center, Geert Grooteplein 10, 6525 GA Nijmegen, The Netherlands; 3 Department of Lipid Biochemistry, Institute of Biochemistry and Biophysics, Polish Academy of Sciences, Pawinskiego 5a, 02-106 Warsaw, Poland

## Abstract

Congenital disorders of glycosylation (CDG) comprise a group of inborn errors of metabolism with abnormal glycosylation of proteins and lipids. Patients with defective protein N-glycosylation are identified in routine metabolic screening via analysis of serum transferrin glycosylation. Defects in the assembly of the dolichol linked Glc_3_Man_9_GlcNAc_2_ glycan and its transfer to proteins lead to the (partial) absence of complete glycans on proteins. These defects are called CDG-I and are located in the endoplasmic reticulum (ER) or cytoplasm. Defects in the subsequent processing of protein bound glycans result in the presence of truncated glycans on proteins. These defects are called CDG-II and the enzymes involved are located mainly in the Golgi apparatus. In recent years, human defects have been identified in dolichol biosynthesis genes within the group of CDG-I patients. This has increased interest in dolichol metabolism, has resulted in specific recognizable clinical symptoms in CDG-I and has offered new mechanistic insights in dolichol biosynthesis. We here review its biosynthetic pathways, the clinical and biochemical phenotypes in dolichol-related CDG defects, up to the formation of dolichyl-P-mannose (Dol-P-Man), and discuss existing evidence of regulatory networks in dolichol metabolism to provide an outlook on therapeutic strategies.

## Biosynthesis of dolichol

All tissues in eukaryotic organisms contain dolichol metabolites. In human, they occur as dolichol (Dol) or dolichyl-phosphate (Dol-P), while also dolichol esters and dolichoic acid have been identified, for example in bovine thyroid (Steen et al 1984; Van Dessel et al 1993) and human brain (Guan 2009). Apart from the well studied localization of Dol-P in the endoplasmic reticulum for protein N-glycosylation, almost all organelle membranes, such as Golgi, mitochondria and lysosomes, contain dolichol metabolites. Very limited knowledge is available on the function of dolichol metabolites in these organelles, like a modulatory effect of Dol and Dol-P on the physico-chemical properties of lipid bilayers as well as a protective ‘shielding’ of cellular lipids against the oxidative damage caused by ROS. Numerous reviews summarizing the literature on the cellular role of dolichol and Dol-P are recommended to interested readers (Chojnacki and Dallner 1988; Bergamini et al 2004; Swiezewska and Danikiewicz 2005; Cantagrel and Lefeber 2011; Surmacz and Swiezewska 2011). In recent years, dolichol metabolism gained considerably increased interest in the context of protein glycosylation due to the identified physiological consequences of disruptions in this process, especially in the congenital disorders of glycosylation (CDG). Identification of such gene defects resulted in significant progress in understanding of the molecular background of dolichol biosynthesis, but still many questions remain unsolved. In this section, we review the current knowledge on the network of enzymatic interactions for production of dolichol and its glycosylated metabolites.

**Fig. 1:**
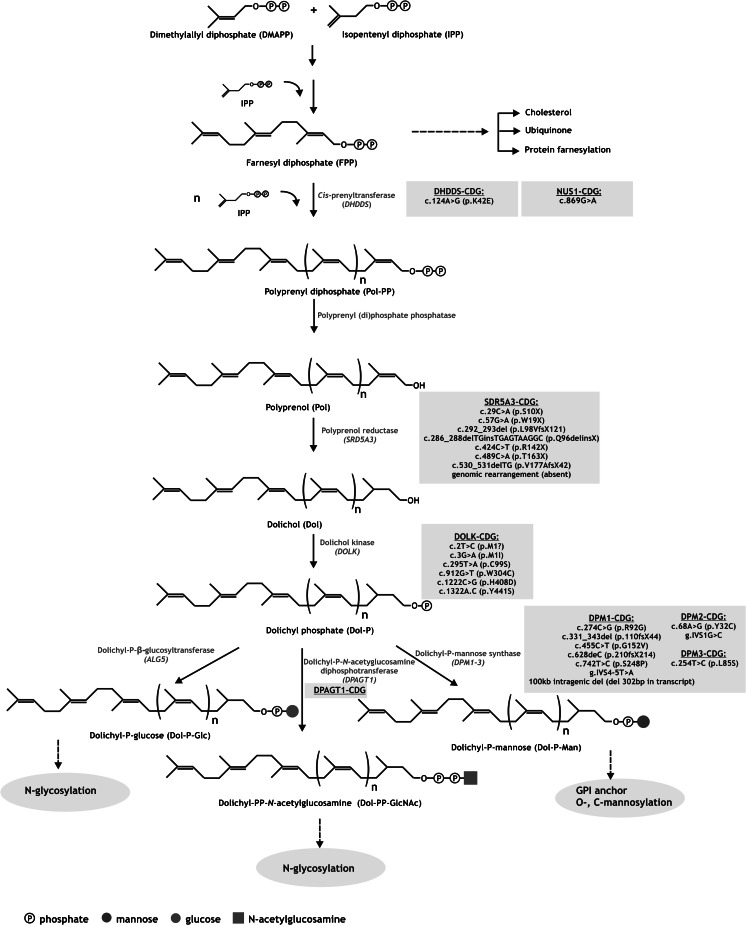
Schematic presentation of the enzymatic network leading to production of dolichol and its glycosylated metabolites. Indicated are the biosynthesis-related genes (in italic), glycosylation pathways (gray ovals) and mutations in the genes causative for particular CDGs (gray boxes). All the enzymes involved in the synthesis of dolichol and its glycosylated derivatives are located in the ER or cytoplasm

The schematic presentation of the biosynthetic pathway leading to the synthesis of Dol-P is shown in Fig. 1. Dolichol in animals and yeast is considered as the end-product of the mevalonate (MVA) pathway. In summary, condensation of three acetyl-CoA molecules gives rise to 3-hydroxy-3-methylglutaryl-CoA which by HMG-CoA reductase (HMGR), the enzyme considered as the regulatory point of the entire MVA pathway, is converted into mevalonate. Combined activity of three subsequent enzymes leads to synthesis of isopentyl diphospate (IPP), the building block for isoprenoids. Further condensation of three IPP molecules results in formation of farnesyl diphosphate (FPP), which is considered as a critical branch-point of the pathway. It serves as substrate for four different pathways: squalene synthase that catalyzes the first step leading to production of cholesterol, *trans*-prenyltransferase involved in ubiquinone side-chain synthesis, protein farnesyltransferase responsible for posttranslational farnesylation of proteins and *cis*-prenyltransferase, the first specific enzyme in dolichol biosynthesis pathway.

*Cis*-prenyltransferase in yeast and animals uses FPP as starter and catalyzes its numerous subsequent condensations with IPP molecules to form polyprenyl diphosphate of a desired chain-length which in fact is a mixture of several homologues. The range of the number of IPPs added is species-dependent; in yeast the dolichol molecules contain 14–18 isoprene units (i.u.) (Quellhorst et al 1998), mammalian cells synthesize longer chains composed of 18–21 units (Rip et al 1985), plant roots produce a broad mixture of homologues, e.g. dolichol of Arabidopsis is composed of 12 to more than 30 i.u. (Jozwiak et al 2013). In contrast, bacterial CPTs produce a single end product which most often contains 11 i.u. Polyprenyl diphosphate is then dephosphorylated by mono and/or diphosphatases (Frank and Waechter 1998) and converted to dolichol by polyprenol reductase (Cantagrel et al 2010) absent from prokaryotic cells. Dolichol is further phoshorylated by a dolichol kinase (Shridas and Waechter 2006) and might then serve as the carrier for mannose and glucose monosaccharides further used as donors in N-glycosylation, O- and C-mannosylation and GPI anchor synthesis. Moreover, Dol-P serves as carrier of theGlcNAc_2_Man_9_Glc_3_ oligosaccharide precursor for protein N-glycosylation. Dol-P-Man is synthesized by Dol-P-Man synthase via the DPM1-3 protein complex in human (Maeda and Kinoshita 2008), while yeast contains a single DPM1 protein. Dolichyl-P-glucose (Dol-P-Glc) is produced by dolichyl-phosphate β-glucosyltransferase (ALG5 in human) (Heesen et al 1994). Synthesis of the DolPP-GlcNAc_2_Man_9_Glc_3_ starts on the cytoplasmic side of the ER and afterwards the intermediate DolPP-GlcNAc_2_Man_5_ is flipped by RFT1 (Haeuptle et al 2008) into the ER lumen where assembly of oligosaccharide chain is completed. Finally, the fully formed oligosaccharide structure GlcNAc_2_Man_9_Glc_3_ is co-translationally transferred to the asparagine residue of the growing polypeptide with release of Dol-PP that is recycled (Kornfeld and Kornfeld 1985; Banerjee 2012). The literature on biosynthesis of the Dol-PP-glycan has been presented in numerous excellent reviews (Denecke and Kranz 2009, Aebi 2013, Breitling and Aebi 2013).

### Molecular mechanisms of dolichol biosynthesis

Human genes encoding enzymes involved in the synthesis of dolichol (Fig. 1) are listed in Table 1.

**Table 1:** Genes encoding enzymes involved in dolichol biosynthesis in human

Gene	Enzyme	Other names	GenBank number	Locus
*DHDDS*	*cis-*prenyltransferase	dehydrodolichyl diphosphate synthase	NM_024887	1p35.3
*DOLPP1*	mono/ diphosphatase	dolichyl pyrophosphate phosphatase 1	NM_020438	9q34.1
*SRD5A3*	polyprenol reductase	steroid 5 alpha-reductase 3	NM_024592.4	4q12
*DOLK*	dolichol kinase		NM_014908	9q34.13

#### Cis-prenyltransferase

CPT, syn. dehydrodolichyl diphosphate synthase, DHDDS, is the first enzyme dedicated exclusively to dolichol biosynthesis. It is a highly conserved enzyme that can be found in all animal species, as well as in plants and bacteria. The biochemical and molecular characteristic of these enzymes have been studied earlier (Liang et al 2002; Takahashi and Koyama 2006). The prokayotic genes encoding *cis*-prenyltranfereses (syn. undecaprenyl diphosphate synthase, UDPS) have been cloned from numerous bacteria, e.g. *Micrococcus luteus* (Shimizu et al 1998), *Escherichia coli* and *Haemophilus influenzae* (Apfel et al 1999). Eukaryotic *cis*-prenyltransferases identified in *Saccharomyces cerevisiae* are encoded by the *RER2* and *SRT1* genes, expression of which is differently controlled during cell growth (Sato et al 1999; Schenk et al 2001b). It was shown that two yeast isoforms demonstrate different subcellular localization and physiological role. Rer2p, constitutively expressed enzyme, is localized to the ER and synthesizes dolichol molecules with 14–17 isoprene units, whereas Srt1p is mainly found in lipid particles (lipid bodies) and produces long chain molecules comprised of 19–22 units similar to mammalian dolichols (Sato et al 1999). Mechanism by which individual CPT enzymes recognizes the prenyl chain lengths of substrates and products are postulated by Takahashi and Koyama (2006).

Through comparison of the yeast *CPT* sequences with plant genomic sequences, a few plant homologous genes have been cloned, e.g. two genes from *Arabidopsis thaliana* (Cunillera et al 2000; Oh et al 2000, Kera et al 2012; Surmacz et al 2013) and the presence of a multiple *CPT* gene families has been confirmed in plants (Surmacz and Swiezewska 2011; Akhtar 2013). Similar analysis of sequence homology revealed a single gene encoding human CPT/DHDDS (Shridas et al 2003; Endo et al 2003). The human enzyme was able to complement the Rer2 yeast deletion mutant. The *hCPT*/*DHDDS* gene comprises eight coding exons (Shridas et al 2003; Zelinger et al 2011), encoding a protein of 334 amino acids (Shridas et al 2003; Endo et al 2003) and was mapped to chromosome 1p36.11.

Overexpression of hCPT in mammalian cells results in only slight increase of its enzymatic activity suggesting that an accessory protein might be required for full activity of human *cis*-prenyltransferase (Shridas et al 2003). This interesting supposition has further been experimentally confirmed and the molecular machinery responsible for regulation of hCIT activity has been partially resolved by Harrisson et al (2011). The authors identified Nogo-B receptor (NgBR) as component modulating CPT activity and regulating dolichol biosynthesis in mammalian cells. Previously, NgBR has been described as a NPC2-binding protein (Nieman-Pick type 2) that promotes NPC2 stability in the ER lumen. It was shown that NgBR exists in two topological conformations. The minor fraction with C-terminal end facing the luminal side of the ER related to regulation of NPC2 stability (Harrison et al 2009) while the major fraction with its C-terminal end oriented towards the cytosol regulates CPT activity and binds hCPT (Harrison et al 2011). Functional studies in the yeast mutant *rer2* revealed that NgBR is not sufficient to rescue its growth phenotype and confirm that this protein is not an independent single subunit enzyme but is rather necessary to maintain the enzymatic activity of hCPT. Loss of NgBR expression results in robust deficit in CPT activity and consequently undetectable levels of dolichol. Co-immunoprecipitation confirmed that the C-terminal domain of NgBR is necessary for its interaction with hCPT (Harrison et al 2009).

#### Polyprenyl (di)phosphate phosphatase

Activity required to dephosphorylate polyprenyl diphosphate has been reported in several biological systems including yeast, mammals and plants (Wedgwood and Strominger 1980; Adair and Cafmeyer 1983; Wolf et al 1991; Ravi et al 1983). The gene encoding this (di)phosphatase activity is still unclear. The *CWH8* gene in *Saccharomyces cerevisiae* encodes a phosphatase capable of converting Dol-PP to Dol-P and also at a slower rate is able to dephosphorylate Dol-P (Fernandez et al 2001). This activity is required after release of Dol-PP from the protein glycosylation reaction for new rounds of glycosylation. Additionally, two other genes encoding Mg^2+^-independent lipid phosphatases, *LLP1* and *DPP1*, have been identified in *Saccharomyces* with hydrolytic activities towards Dol-PP, Dol-P, farnesyl-PP and geranylgeranyl-PP (Faulkner et al 1999). *DOLPP1*, the mammalian ortholog of yeast *CWH8* shares a high degree of sequence identity with the yeast enzyme (Rush et al 2002) and is able to complement defects in growth, protein N-glycosylation and accumulation of Dol-PP in the *cwh8* mutant yeast cells. Mouse polyprenyl phosphate phosphatase is comprised of 238-amino acids, contains four putative transmembrane domains and possesses a consensus lipid-phosphate phosphatase motif. The N- and C- termini are predicted to be on the cytosolic face of the ER, and the catalytic domain is predicted to be on the luminal face of the ER (Rush et al 2002). Radiation hybrid analysis and FISH revealed that human *DOLPP1* (also termed *LSFR2*) is located on chromosome 9q34.1 (Gilley and Fried 1999) and comprises eight exons. No details are available on its structure-function studies in human cells.

#### Polyprenol reductase

Polyprenol reductase produces dolichol by reduction of the α-terminal isoprene residue of polyprenol. Biochemical characterization of polyprenol reductase of rat liver and yeast has been presented earlier (Sagami et al 1993; Tateyama and Sagami 2001), while the first identification of the gene encoding polyprenol reductase has been achieved in human by Cantagrel et al (2010). They showed that SRD5A3 is essential for protein N-linked glycosylation in mammals (Cantagrel et al 2010). The yeast polyprenol reductase is encoded by the *DFG10* gene. Expression of human *SRD5A3* in the *dgf10* mutant yeast cells rescued the phenotype of carboxypeptidase Y (CPY) underglycosylation and partially decreased the accumulation of polyprenols. In Arabidopsis, the presence of two *SRD5A3-LIKE* genes has been postulated (Jadid et al 2011; Jozwiak et al unpublished data).

In human, a single gene encoding polyprenol reductase is located on chromosome 4p12 and contains five exons encoding a protein of 318 amino acids. The SRD5A3 protein has six transmembrane domains and is localized in the endoplasmatic reticulum (ER) and endoplasmic reticulum-Golgi intermediate compartment (ERGIC). The structure of polyprenol reductase has not been described yet. SRD5A3 has been considered so far as a member of the 5α-steroid reductase family and its involvement, along with SRD5A1 and −2, in reduction of testosterone to dihydroteststerone is shown in several systems (Uemura et al 2008; Mitsiades et al 2012; Titus et al 2014). However, the recent data on SRD5A3 function in polyprenol reduction questions the role of steroids as endogenous substrates.

Analysis of *SRD5A3* expression identified the transcript of this gene in virtually all human tissues: brain, kidney, liver, stomach, pancreas, hearth, placenta, spleen, duodenum, colon, breast, thyroid, tonsil, skin and also cancer cell lines (Kazutoshi et al 2008; Uemura et al 2008; Morava et al 2010; Godoy et al 2011; Azzouni et al 2012). The hydrophobic N-terminal region, probably responsible for interaction with the substrates, and the H296 residue located in the catalytic domain of SRD5A3 could have critical roles for its androgen-producing activity, since this activity is abolished by mutations at both sites (Uemura et al 2008). Homozygous nonsense mutations in the *SRD5A3* gene leading to early truncated protein were found in SRD5A3-CDG patients. This resulted in polyprenol accumulation and normal or increased levels of dolichols in plasma and tissues of patients (Cantagrel et al 2010). Reduced levels of dolichol and its derivative Dol-P are supposed to affect N-glycosylation of proteins, suggesting that only the local pool of Dol-P in the ER is limiting, and results in a congenital disorders of glycosylation type I (CDG I) (Cantagrel et al 2010; Lefeber et al 2011). Clearly, an alternative pathway for dolichol synthesis must be present in yeast, mouse and human to account for residual dolichol in these null-mutants. Potentially, the oxidative pathway involving dolichal could play such a role (Sagami 1996). It has been shown that deletion of the *SRD5A3* gene in mice disrupted protein glycosylation and resulted in death of mouse embryos at embryonic day 12.5 (Cantagrel et al 2010). SRD5A3 involvement in cellular processes has recently been reviewed (Cantagrel and Lefeber 2011; Azzouni et al 2012; Traish 2012).

#### Dolichol kinase

Dolichol kinase catalyzes the CTP-mediated phosphorylation of dolichol (Fernandez et al 2002). In yeast, dolichol kinase is encoded by *SEC59* (Heller et al 1992). One gene encoding a putative dolichol kinase was found in the *Arabidopsis thaliana* genome [BLASTP screen], while biochemical characterization of DOLK has been performed in soybean (Rip and Carroll 1988). A bacterial enzyme was purified from *Lactobacillus* (Kalin and Allen 1979). Human dolichol kinase has been characterized at the molecular level by the group of Waechter (Fernandez et al 2002; Shridas and Waechter 2006). Human *DOLK (DK1)* is located on chromosome 9 (Kikuno et al 1999). The open reading frame encodes a protein with 538 amino acids. Highest expression levels of *DOLK* mRNA was observed in fetal and adult brain, followed by fetal skeletal muscle and heart and adult heart tissue (Lefeber et al 2011). Dolichol kinase is a hydrophobic protein with 13 membrane-spanning domains (Shridas and Waechter 2006). However, Haeuptle and Hennet (2009) predicted 15 transmembrane domains. Structure-function studies indicate that the C-terminal domain exposed to the cytoplasmic face contains a CTP-binding pocket. Deletion of this region or mutation of conserved residues (K470/471 and T472) within this motif results in a partial or total loss of activity and altered affinity for CTP. What is more, G320D substitution in the mutated enzyme of yeast cells was found. Conversion of the corresponding residue G443D in human dolichol kinase abolish its activity (Shridas and Waechter 2006).

### Dolichol cycle

By phosphorylation of dolichol, the dolichol kinase enables the transfer of monosaccharides to Dol-P and the initiation of dolichol-linked oligosaccharide biosynthesis. This process takes place on both sites of the ER membrane and requires the activity of several specific glycosyltransferases. The monosaccharides added to the growing lipid linked oligosaccharide (LLO) are derived from nucleotide-activated donors or from Dol-P-linked monosaccharides. UDP-GlcNAc and GDP-Man serve as substrates for the transferases that act on the cytoplasmic side of the ER membrane, whereas Dol-P-linked sugars are substrates for lumenal glycosyltransferases. Since enzymes participating in the synthesis and modification of the LLO and respective genes have been described in numerous excellent reviews (e.g. Haeuplte and Hennet 2009; Banerjee 2012; Aebi 2013; Breitling and Aebi 2013), only a brief summary on selected steps of the LLO pathway is presented below.

Dol-P-Man is utilized as a substrate for N-glycosylation, O-mannosylation of proteins (Lommel and Strahl 2009) and biosynthesis of GPI-anchored proteins (Orlean and Menon 2007). Dol-P-Man is generated from Dol-P and GDP-mannose by the Dol-P-Man synthase, which in humans forms an oligomeric enzyme complex comprised of DPM1, DPM2 and DPM3 (Maeda et al 2000). DPM1 is the catalytic component associated with DPM3 and DPM2 that is required to target cytosolic DPM1 to the ER membrane. The structural characteristics of the mammalian Dol-P-Man synthase complex and the roles of individual subunits has been described (Maeda and Kinoshita 2008).

It is also worth noting that MPDU1, a membrane protein localized in the ER, is essential for the use of Dol-P-Man and Dol-P-Glc in GPI and LLO biosynthesis and protein O-, C-mannosylation although its precise function is unclear (Anand et al 2001).

Monosaccharides and oligosaccharide chains carried on Dol-P are necessary for different glycosylation pathways and require sufficient amounts of Dol-P. To provide an adequate cellular concentration of Dol-P, *de novo* synthesis is assisted by the recycling pathway. An essential step in this recycling is the conversion of Dol-PP, released by the oligosaccharyltransferase, to Dol-P by diphosphatase that acts at the luminal side of the ER. In yeast, this reaction is catalyzed by the CWH8 phosphatase and in human by DOLPP1. It is as yet unclear if DOLPP1 can also dephosphorylate polyprenyl-PP during polyprenol synthesis. An additional appealing issue for clarification is how Dol-P flips across the ER membrane for new rounds of Dol-PP-glycan synthesis, because no potential Dol-P flippase has been described to date. A potential mechanism for translocation of Dol-P is discussed by Breitling and Aebi (2013).

## Dolichol-related human disease phenotypes

As discussed above, dolichol and its metabolites perform pleiotropic functions in cellular systems and the clinical symptoms that result from genetic deficiencies in its biosynthesis are not easily predicted. The identification of dolichol biosynthesis defects within the group of CDG-I patients has allowed us to start correlating clinical symptoms with biochemical pathways. The classical presentation of CDG-I defects is a multi-organ disease with neurological involvement. A few subtypes without neurological symptoms, such as MPI-CDG, have also been observed. The metabolic pathway required for synthesis of the Glc_3_Man_9_GlcNAc_2_-PP-dolichol precursor in the ER is identical in each eukaryotic cell and, as a dogma, all N-glycosylated proteins are similarly affected resulting in a multisystem disease. In practice, the situation is more subtle with some proteins being more severely affected than others. For example, lack of complete N-glycans on intercellular adhesion molecule-1 (ICAM1) result in its absence on the plasma membrane, whereas other proteins can be normally secreted but have defective function. The consequences are different for a defect in sugar supply pathways, such as the availability of the sugar donor Dol-P-Man, as these defects are not restricted to N-glycosylation. Other glycosylation pathways executed in the ER including C- and O-mannosylation, GPI-anchor biosynthesis and O-glucosylation also depend on available dolichol. In addition, other cellular functions depending on the availability of dolichol metabolites might be disturbed. In this section, we will review the currently known genetic diseases in such ”shared” pathways and discuss the current understanding of specific clinical symptoms in relation to different glycosylation pathways, especially dystroglycan O-mannosylation (we also refer to previous reviews of Denecke and Kranz 2009; Cantagrel and Lefeber 2011 and Wolfe et al 2012). Table 2 summarizes characteristic clinical symptoms that can occur (more or less) isolated or in combination with other symptoms in a multisystem presentation.

**Table 2:** Diagnostic clinical clues in dolichol cycle defects

Characteristic symptoms	Gene
Ichthyosiform skin	*MPDU1, DOLK, SRD5A3*
Dilated cardiomyopathy	*DOLK, DPM3*
Muscle dystrophy and/or increased CK	*DPM1, DPM2, DPM3*
Non-syndromal retinitis pigmentosa	*DHDDS*
Ocular abnormalities (coloboma, glaucoma, cataract, optic atrophy)	*SRD5A3*

### DHDDS-CDG (MIM 613861)

*Cis*-prenyltransferase (DHDDS) is the first commited step in Dol-P biosynthesis. Thus far, no patients with abnormal N-glycosylation have been reported with a defect in DHDDS. However, the K42E and T206A missense mutations have been linked to autosomal recessive nonsyndromal retinitis pigmentosa (RP) (Zelinger et al 2011; Zuchner et al 2011; Wen et al 2013). In plasma and urine of patients, a characteristic shortening of dolichols was identified by mass spectrometry (Wen et al 2013). Instead of the common dolichol-19 species, dolichol-18 was the dominant species in patients. Interestingly, no significant abnormality in protein glycosylation has been observed of plasma transferrin in deficient patients. Suppression of *DHDDS* expression in zebrafish leads to the loss of photoreceptor outer segments and visual function. These observations support the hypothesis that insufficient DHDDS function leads to retinal degeneration (Wen et al 2014). Still the cellular mechanisms explaining whether and how the shortened dolichol profiles contribute to the retinal degeneration phenotype awaits clarification.

### NUS1-CDG (no MIM entry yet)

NUS1, encoding the NogoB receptor is essential for cis-prenyltransferase activity and is known to bind to the NPC2 protein, involved in cholesterol metabolism. Recently, a homozygous missense mutation was identified in NUS1 in two siblings (Park et al 2014). Patients presented with scoliosis, severe epilepsy, muscle hypotonia, developmental delay, microcephaly, and visual impairment. Cholesterol in plasma was normal but increased in patient fibroblasts. In fibroblasts, incorporation of mannose in glycoproteins was reduced and LAMP1 and ICAM1 seemed to be hypoglycosylated. In plasma and urine, the dolichol chain length shifted to shorter lengths in a patient and also slightly in two analyzed heterozygous carriers, with dolichol-19 being most abundant in controls and dolichol-18 in the patient. No mention was made of dilated cardiomyopathy, increase of creatine kinase or skin involvement, as seen in other CDG subtypes discussed in this review.

### SRD5A3-CDG (MIM 612379)

Polyprenol reductase is needed for conversion of polyprenol to dolichol. Mutations in steroid 5α-reductase type 3 encoding gene (*SRD5A3* **)** lead to a purely neurological disease with developmental delay, ataxia and early visual impairment with optic atrophy (Cantagrel et al 2010; Morava et al 2010). Prominent ocular abnormalities were observed with retinal coloboma, congenital cataract and glaucoma. In addition, ichthyosiform dermatitis was reported with liver dysfunction and coagulation abnormalities. None of the patients presented with muscle or heart pathology. Two adult patients (∼40 years old) were recently described with an isolated presentation of ataxia (Kara et al 2014).

Analysis of polyisoprenoids in SRD5A3-CDG patient plasma by mass spectrometry (Cantagrel et al 2010) revealed an increase of the concentration of polyprenols, which facilitates diagnosis. In patient cells, no clear abnormalities could be observed of polyprenols and dolichols. In *dfg10* knock-out yeast, however, polyprenol accumulation was observed with a concomitant considerable decrease in levels of dolichol. Interestingly, irrespective of early truncating mutations in all patients, dolichol was still present in plasma and cells of patients. This lead to the suggestion of the presence of an additional protein involved in polyprenol reduction (Cantagrel et al 2010). It could well be that the presence of such an alternative pathway explains the absence of muscle dystrophy and/or dilated cardiomyopathy in SRD5A3-CDG patients.

### DOLK-CDG (MIM 610768)

Dolichol kinase is required for phosphorylation of dolichol. Dol-P serves as precursor for synthesis of Dol-P-Man by Dol-P-Man synthase and Dol-P-Glc by ALG5 but also serves as anchor for the Glc_3_Man_9_GlcNAc_2_ glycan. The first description of four patients with mutations in *DOLK* (Kranz et al 2007) revealed a very severe phenotype with early death. Characteristic symptoms were dilated cardiomyopathy, muscular hypotonia, and ichthyosiform abnormalities of the skin. Involvement of Dol-P in multiple pathways, additionally to glycosylation, was suggested to be related to the peculiar clinical phenotype. A subsequent study (Lefeber et al 2011) revealed a milder course of disease in 11 patients with a presentation dominated by dilated cardiomyopathy. In addition, ichthyosis was noted in several patients without significant muscular weakness or elevation of creatine kinase. In heart muscle, abnormal O-mannosylation of alpha-dystroglycan was found, which was suggested to be correlated with the occurrence of dilated cardiomyopathy (DCM). Two additional case reports showed a multisystem presentation, including DCM, epilepsy and dysmorphic features (Lieu et al 2013) in one patient and a purely neurological presentation in a sib-pair (Helander et al 2013) without the occurrence of heart or skeletal muscle problems.

### DPM-CDG (DPM1: MIM 608799 ; DPM2: MIM 615042 ; DPM3: MIM 612937)

Dol-P-Man synthase is required for synthesis of Dol-P-Man and is composed of three subunits (DPM1-3). Early fatal disease causing mutations were first described for the catalytic subunit DPM1 (Imbach et al 2000; Kim et al 2000). Patients presented with severe congenital visual loss, optic atrophy and seizures. A milder course was later observed in a 9-year old patient with mild dysmorphic features, developmental delay, microcephaly, optic atrophy, cerebellar dysfunction and elevated creatine kinase (Garcia-Silva et al 2004). Dancourt et al (2006) reported a brother and sister with a presentation dominated by ataxia, with normal creatine kinase. The overall presentation of DPM1-CDG was interpreted as the characteristic multisystem phenotype of CDG-I defects. In 2009, a homozygous mutation was identified in *DPM3* in a young woman who presented with muscle weakness due to muscular dystrophy as diagnosed after muscle biopsy and a dilated cardiomyopathy (Lefeber et al 2009). These particular symptoms were not known for CDG-I N-glycosylation defects. Further studies revealed abnormal O-mannosylation of alpha-dystroglycan in a muscle biopsy, which linked CDG with the dystroglycanopathies, a subgroup of the congenital muscular dystrophies. Patients with a dystroglycanopathy present with muscle dystrophy, while dilated cardiomyopathy has been reported in several milder cases (Pane et al 2012). Expression of the GPI-anchored protein CD59 on fibroblasts and C-mannosylation of serum properdin were normal. DPM2-CDG (Barone et al 2012) was described in three infantile patients with profound developmental delay, intractable epilepsy and severe hypotonia with elevated blood creatine kinase levels. Abnormal O-mannosylation was confirmed in a muscle biopsy. For DPM1-CDG, abnormal O-mannosylation in muscle was recently described (Yang et al 2013) in a patient with severe motor delay and elevated creatine kinase. Although it cannot be excluded that other glycosylation pathways are also affected and contribute to the clinical presentation in more severely affected patients, the current data suggest that the clinical symptoms in Dol-P-Man synthase defects are correlated with abnormal N-glycosylation and O-mannosylation.

### MPDU1-CDG (MIM)

MPDU1 is required for utilization of the Dol-P-Man and Dol-P-Glc donors in ER glycosylation reactions, although the exact mechanism of action remains unsolved. Only very few patients with MPDU1-CDG have been reported (Schenk et al 2001a; Kranz et al 2001). Patients presented with psychomotor retardation, epilepsy and skin disease, including ichthysosis.

### Outlook to new genes and CDG subtypes

Identification of novel gene defects in dolichol metabolism has already provided novel insights in biochemistry and clinical symptoms, but novel findings are to be expected. More severe mutations in DHDDS could very well result in a multisystem phenotype, including symptoms of CDG-I, ichthyosis and the dystroglycanopathies and an abnormal type I transferrin isofocusing profile. For DOLPP1, no genetic defect has been reported to date. Its role is primarily located in the recycling of Dol-P after N-glycosylation, which would suggest a likely multisystem phenotype as in other CDG-I defects. Nevertheless, this could as well result in diminished levels of Dol-P as in DOLK-CDG, which could give rise to clinical symptoms such as muscle dystrophy, ichthyosis and dilated cardiomyopathy. The occurrence of ichthyosis is limited to the dolichol biosynthesis defects SRD5A3-CDG and DOLK-CDG, but also occurs in MPDU1-CDG and not in DPM-CDG. This might hint to a direct role of MPDU1 in lipid metabolism. Possibly, this could include the utilization of Dol-P apart from its confirmed role in utilization of Dol-P-Man and Dol-P-Glc (Anand et al 2001). Finally, for some of the biochemical steps in dolichol metabolism, genes still have to be identified, for example the possible flipping of Dol-P from ER to cytosol, the dephosphorylation of polyprenyl-PP during biosynthesis and the alternative pathway for polyprenol reduction. Identification of such additional gene defects in dolichol metabolism and detailed clinical characterization will help to solve issues like tissue specific biochemical mechanisms and the flux through dolichol biosynthesis towards different glycosylation pathways.

## Opportunities for overcoming defects in dolichol biosynthetic pathways

A facile approach towards therapeutic strategies in dolichol cycle defects would be to replenish the missing substrates dolichol or Dol-P (Jakobsson et al 1989; Kalen et al 1990). However, the contribution of dietary dolichol metabolites to the cellular pool of dolichol for glycosylation reactions seems to be very limited, as shown experimentally and reviewed (Cantagrel et al 2011). Strategies to target dolichols to the site of glycosylation in the ER would circumvent this problem. Although most of the biosynthetic steps have been characterized at the molecular level, knowledge about the regulation of particular enzymes remains scarce with one exception, namely HMGR, the most studied enzyme in literature. Additionally, nothing is known about networking and global regulation of the dolichol pathway. In this section, we aimed to summarize the literature on regulatory mechanisms modulating the activity of dolichol synthesizing enzymes and consequently affecting the cellular levels of dolichol metabolites, which might provide future opportunities to improve dolichol availability for protein glycosylation.

### Regulation of *cis*-prenyltransferase (CPT)

Data on the factors regulating activity of CPT are limited. So far only a few observations have been published and no regulatory network has been suggested.

A pleiotropic stimulator cAMP increases the activity of human *cis*-prenyltransferase as shown by a tenfold increase of CPT activity in human placenta choriocarcinoma (JEG-3) cells, pretreated with 8-bromo-cAMP. This is in line with a 60- and 20-fold enhancement of *de novo* synthesis of dolichol and dolichyl phosphate, respectively. These results have been substantiated by the induction of hCPT activity in a cAMP-generating system (activation of adenylate cyclase by forskolin in the presence of phosphodiesterase inhibitor IBMX). Stimulation of the dolichol/Dol-P pathway by cAMP seems specific since biosynthesis of cholesterol has not been stimulated upon the same conditions (Konrad and Merz 1996). A more recent study implicates protein kinase PKA type I in the modulation of cAMP-mediated regulation of protein N-glycosylation. Moreover Dol-P-Man synthase is postulated as the main target of PKA I (Banerjee 2007).

CPT activity is modulated in response to developmental changes since Dol-P level increases linearly through the cell cycle in mouse L-1210 cells, reaching twofold increase in S phase in comparison to early G1. Consistently, *cis*-prenyltransferase activity is increasing through G1 to a maximum level in the S phase (Adair and Cafmeyer 1987). Likewise, a concomitantly increased activity of rat CPT and LLO synthesis was noted in the S phase of synchronized cultures of rat 3Y1 cells (Fukushima et al 1997). Finally, *in vitro* CPT activity measurement using rat liver microsomes reveals a low level after birth which increases continuously up to day 54 and then decreases to a low level maintained throughout the remainder of the study (365 days). Simultaneous monitoring of dolichol concentrations in rat liver indicates a continuous increase throughout the entire period in contrast to the constant level of cholesterol (Thelin et al 1994).

On the other hand, an age-related accumulation of dolichol is postulated to be linked to a higher availability of mevalonate, which is linked to an increased activity of HMGR due to the loss of enzymatic regulation (Pallottini et al 2003).

A large increase of CPT activity (15-fold) and increased Dol-P synthesis is noted during the proliferative response of murine B lymphocytes (B cells) to bacterial lipopolysaccharide (LPS) (Crick et al 1994).

Modulation of expression of CPT encoding genes has been observed in plants. Transcription and activity of LlCPT (identified in *Lilium longiflorum anther-66*) is regulated during microspore development in the anther, while additionally the expression of *LLA66* is regulated by plant hormones, gibberellin and ethylene (Liu et al 2011). Increased expression of *AtCPT6* (also called heptaprenyl diphosphate synthase AtHEPS) is caused by cold treatment of Arabidopsis seedlings (Kera et al 2012). Sugar type (glucose or sucrose ) and concentration modulate the expression level of all six AtCPT encoding genes detected in *A. thaliana* hairy roots (Jozwiak et al 2013).

A few interesting but not fully explored observations indicate the involvement of various genes/proteins in regulation of CPT activity. This might shed light on the cellular network of CPT in diverse biological systems. For example, an interaction was detected of hCPT with the Niemann–Pick type C2 protein (NPC2) via a yeast two-hybrid screen, which was confirmed by *in vitro* co-immunoprecipitation experiments. This suggested a putative involvement of NPC2 in regulation of hCPT and dolichol biosynthesis (Kharel et al 2004). Nogo-B receptor (NgBR), known to bind NPC2 (Harrison et al 2009), was suggested as modulator of CPT activity in mammalian cells (Harrison et al 2011) — see above for details. *In vitro* studies using rat liver microsomes show that rCPT activity is stimulated sevenfold in the presence of sterol carrier protein-2 with no appreciable effect on polyprenol reductase, dolichol kinase or dolichyl phosphate phosphatase (Ericsson et al 1991).

Studies in *Saccharomyces cerevisiae* revealed a putative role of YTA7 in the dolichol biosynthetic pathway since deletion of YTA7 affected the enzymatic activity of *cis*-prenyltransferase and the cellular levels of isoprenoid compounds (Kuranda et al 2009).

### Regulation of dolichol kinase, dolichyl phosphate phosphatase and polyprenol reductase

First evidence for the developmental regulation of ***dolichol kinase*** activity originate from the finding of twofold increased activity in prenatal pig brains as compared to adult animals with unchanged activity of dolichyl phosphate phosphatase (Scher et al 1985). As mentioned for CPT, also the activity for dolichol kinase and dolichyl phosphate diphosphatase showed cell-cycle dependence (Adair and Cafmeyer 1987). Additionally, environmental factors were found to modulate Dol-P levels. Ethanol treatment resulted in decreased levels of Dol-P (approx. 80% of the control) but did not alter *DOLK* transcript levels in HepaRG cells (Welti and Hülsmeier 2014). In line with decreased Dol-P levels, a smaller fraction of the final oligosaccharide precursor Dol-PP-GlcNAc_2_Man_9_Glc_3_ (77% of the control) has been observed (Welti and Hülsmeier 2014). Estrogens induce dolichol kinase activity due to an increased level of the DOLK enzyme (Burton et al 1981). Finally, dolichol kinase from rat brain and protozoa *Tetrahymena pyriformis* was shown to be regulated by a system involving calmodulin/Ca^2+^ (Gandhi and Keenan 1983).

#### Polyprenol reductase

Data on regulation of polyprenol reductase SRD5A3 are very limited. It was shown that androgen (dihydrotestosterone) regulates the mRNA level of SRD5A1, −2 and −3 isoenzymes in a cell type–specific manner. It was shown that regulation occurs at the transcriptional level and that androgen receptor is recruited to a negative androgen response element (nARE) on the promoter of *SRD5A3 in vivo* and directly binds to the nARE *in vitro* in LNCaP prostate cancer cells (Li et al 2011). This observation is consistent with SRD5A3 overexpression noted in hormone-refractory prostate cancers in which the androgen level is low (Uemura et al 2008). These data, however, do not confirm the role of SRD5A3 in the biosynthesis of androgens.

Modulation of the polyisoprenoid profile upon particular physiological conditions has been observed in yeast and plants. An increased concentration of long-chain polyprenols rather than dolichols was noted in *S. cerevisiae* mutants (mutations in genes encoding farnesyl diphosphate synthase or squalene synthase) for which a significant increase of CPT activity in the late logarithmic phase was observed. Upon such circumstances, polyprenol reductase seems to become inefficient (Szkopinska et al 2006).

Recently, modulation of the ratio of polyprenols versus dolichols has been noted in response to sugar in Arabidopsis hairy root cultures. In the family of long-chain polyisoprenoid alcohols (Dol/Pren-23 dominating), polyprenols are dominating at 3% sucrose and dolichols at 2% glucose (Jozwiak et al 2013). These observations, although indirect, indicate the existence of a cellular machinery responsible for regulation of polyprenol reductase in response to various stimuli.

### Regulation of LLO synthesis

The availability of Dol-P in the ER is considered as a rate-limiting factor in Dol-PP-glycan biosynthesis since an increase in CPT activity and enhanced rate of *de novo* Dol-P biosynthesis increases the capacity of LLO synthesis in embryonic rat brain cells (Crick and Waechter 1994).

Regulatory effects of Dol-P-Man on Dol-PP-GlcNAc synthesis have been observed in several systems (e.g. Kean 1982; Kaushal and Elbein 1985; Shailubhai et al 1988; Kean et al 1994). More recently, a mutual stimulatory effect of Dol-P-Man and Dol-PP-GlcNAc on their synthesis has been observed in the retinas of embryonic chickens (Kean et al 1999). Moreover, feedback inhibition in the formation of Dol-PP-GlcNAc was found by the second intermediate of the pathway, Dol-PP-GlcNAc-GlcNAc. Finally, product inhibition by Dol-PP-GlcNAc itself has been observed (Kean et al 1999).

Another regulatory factor in LLO biosynthesis involves phosphorylation of Dol-P-Man synthase. In *S. cerevisiae*, it was shown that Dol-P-Man synthase activity is regulated by the cAMP-dependent protein kinase A via phosphorylation of S141 (Banerjee et al 2005). Similarly, PKA stimulates Dol-P-Man synthase activity in mammalian cells (Martinez et al 2006; Baksi et al 2008). In line with these observations defective glycosylation is observed in protein kinase A deficient Chinese hamster ovary cells (Banerjee et al 2004).

The signaling role of mannose-6-phosphate in LLO degradation was shown in mouse embryonic fibroblasts (Gao et al 2011).

## Conclusions

The current information on dolichol metabolism as outlined above clearly shows many gaps in our knowledge. Several known genes still have to be linked to human disease phenotypes and further studies on unsolved CDG-I patients could potentially unravel some of the missing genes in dolichol synthesis. Nevertheless, progress in recent years on human genetic disease has revealed novel mechanistic insights. The regulatory aspects in the dolichol pathway and its interconnection in protein networks are still far from being understood. The fragmented information from various model systems precludes the mechanism-based interference in dolichol biosynthesis defects. Analytical advances have revealed useful biomarkers of abnormal dolichol metabolites in plasma and/or urine of SRD5A3-CDG, DHDDS-CDG, and NUS1-CDG patients. Together with the current recognition of specific clinical symptoms related to individual gene defects, this greatly facilitates diagnosis of this group of human defects. Moreover, this will allow the monitoring of medication responses, if mechanism-based studies will lead to novel treatments in the future.
